# Unveiling the Unknown History of the First Civil Hospital of Athens, "Elpis"

**DOI:** 10.7759/cureus.72526

**Published:** 2024-10-28

**Authors:** Spyros N Michaleas, Theodora Psaltopoulou, Panagiotis Halvatsiotis, Ioannis Nikolakakis, Ioannis Dimitriadis, Marianna Karamanou

**Affiliations:** 1 Department of History of Medicine and Medical Ethics, National and Kapodistrian University of Athens School of Medicine, Athens, GRC; 2 Department of Clinical Therapeutics, National and Kapodistrian University of Athens School of Medicine, Athens, GRC; 3 2nd Department of Propaedeutics of Internal Medicine, "Attikon" University General Hospital, National and Kapodistrian University of Athens School of Medicine, Athens, GRC

**Keywords:** epidemics, greece, healthcare, history of medicine, hygiene measures

## Abstract

Until the Othonian University (later known as the National and Kapodistrian University of Athens) began operating, only the Military Hospital and the Maternity Hospital were in operation in Athens. In the mid-18th century, the Elpis General Hospital of Athens was founded as a Civil Hospital, originally situated on Akadimias Street in the building where the Cultural Center of Athens is now located. The Hospital’s current complex in the Ampelokipi area of Athens was initially entrusted to the Hellenic Red Cross to care for war casualties. It functioned as a military hospital until the military decided to vacate the premises and relocate in that area the Municipal Hospital of Athens “Elpis”. Today, the Elpis General Hospital of Athens is equipped with modern facilities and staffed by highly skilled medical and support personnel, continuing to serve the health needs of the public.​

## Introduction and background

This study was conducted as part of postdoctoral research at the Medical School of the National and Kapodistrian University of Athens.

**Figure 1:**
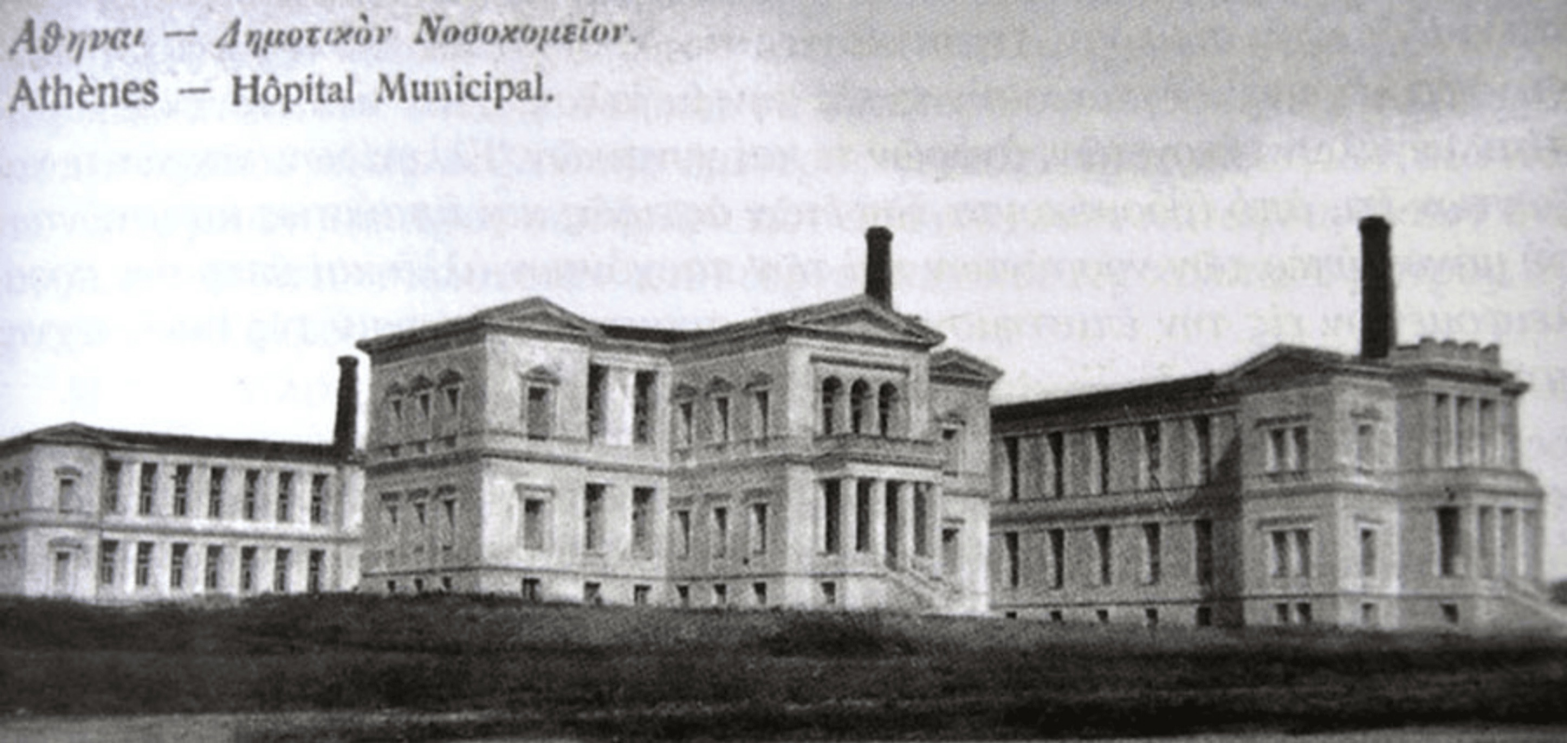
The new building of the hospital in Ampelokipi (1910) Image Source: https://www.elpis.gr; This is an open-source image reproduced under the Creative Commons license

In the capital of the newly formed Greek state, Athens, the only nursing institutions that operated before the founding of the University of Athens in 1837, were the Military Hospital, established in 1836, and the Maternity Hospital, which began operating in 1835. Founded in 1836 as a civil hospital and serving as the Municipal Hospital of Athens “Elpis” from 1842 to 1986, the Elpis General Hospital of Athens remains an important healthcare institution in general medicine and social care (Figure 1). Elpis Hospital was more than just a medical institution; it was a reflection of Athens' struggles and triumphs. Throughout its history, the hospital treated the wounded of the Balkan Wars (1912-1913), supported refugees arriving in Athens during the Interwar period (1922-1940), provided aid during the great famine of the German Occupation (1941-1944), and it was a central figure during the December events (1944) and the Civil War (1946-1949). Its legacy is deeply connected with the history of Athens and the Greek State.

The events of internal and external politics, especially in the difficult and turbulent years of the 19th and the first half of the 20th century, directly or indirectly affected the course and evolution of the hospital institution. For many decades, the Municipal Hospital was the only hospital in the capital, besides the military hospital, that exclusively treated military personnel, symbolizing hope for the people, as its name "Elpis" meaning "Hope" (ἐλπίς) in Greek [1].

Despite facing significant challenges, including shortages of both scientific and auxiliary personnel, as well as limited scientific resources, Elpis Hospital achieved notable advancements. Its enduring operation contributed to the development of modern healthcare practices in Greece, impacting general medicine, infectious disease control, and emergency care. The hospital’s infrastructure underwent several architectural transformations to accommodate the evolving needs of the city’s population and the demands of medical advancements [1].

The hospital’s social impact extended beyond healthcare. It played a vital role in shaping public health policy and fostering the development of medical knowledge. As an institution, it contributed to the social fabric of Athens, offering not only medical assistance but also social services during times of crisis. The initial visionary behind the establishment of the hospital was the doctor, politician, and first mayor of Athens, Anargyros Petrakis (1793-1876). At the hospital, many prominent figures of medical science of Greece began their medical careers, later participating in the establishment of several of the newer hospitals in Athens, such as Theodoros Aretaios (1829-1893), Spyridon Manginas (1839-1920), Nikolaos Makkas (1847-1935), Marinos Geroulanos (1867-1960), and Nikolaos Alivizatos (1876-1945) [1].

## Review

The establishment of Elpis General Hospital of Athens

**Figure 2:**
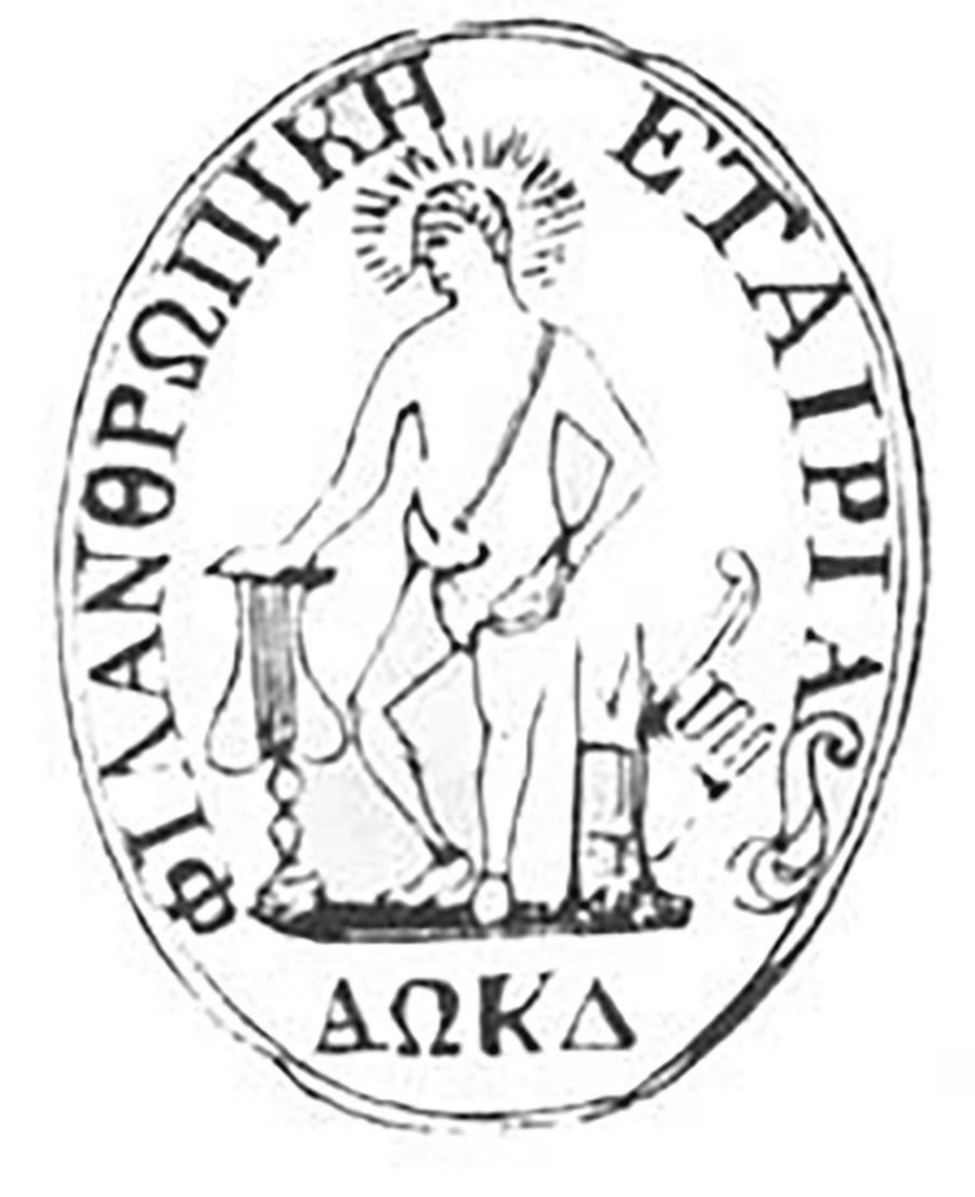
The seal of the Philanthropic Society Image Source: https://argolikivivliothiki.gr; This is an open-source image reproduced under the Creative Commons license.

Among the priorities of the Nafplion Philanthropic Society (Figure 2), founded in 1824 in Nafplio, with branches in Athens and Messolonghi, was the provision of hospital care. In Athens, the local committee of the society consisted of two Athenians, Anargyros Petrakis (1793-1876) and Georgios Psyllas (1794-1878), and an Italian, DK Vitalis. After the liberation of Athens and the declaration of Greece as an independent state (London Protocol, February 3, 1830) [2], Petrakis was elected to the city’s temporary alderman, while the scholar Psyllas was appointed Prefect (October 12, 1833). The first action of the Philanthropic Society was the issuance of a proclamation, on October 8, 1824, to the residents of Athens, which, among other things, emphasized the importance of social solidarity and the significance of hospital treatment [1].

**Figure 3:**
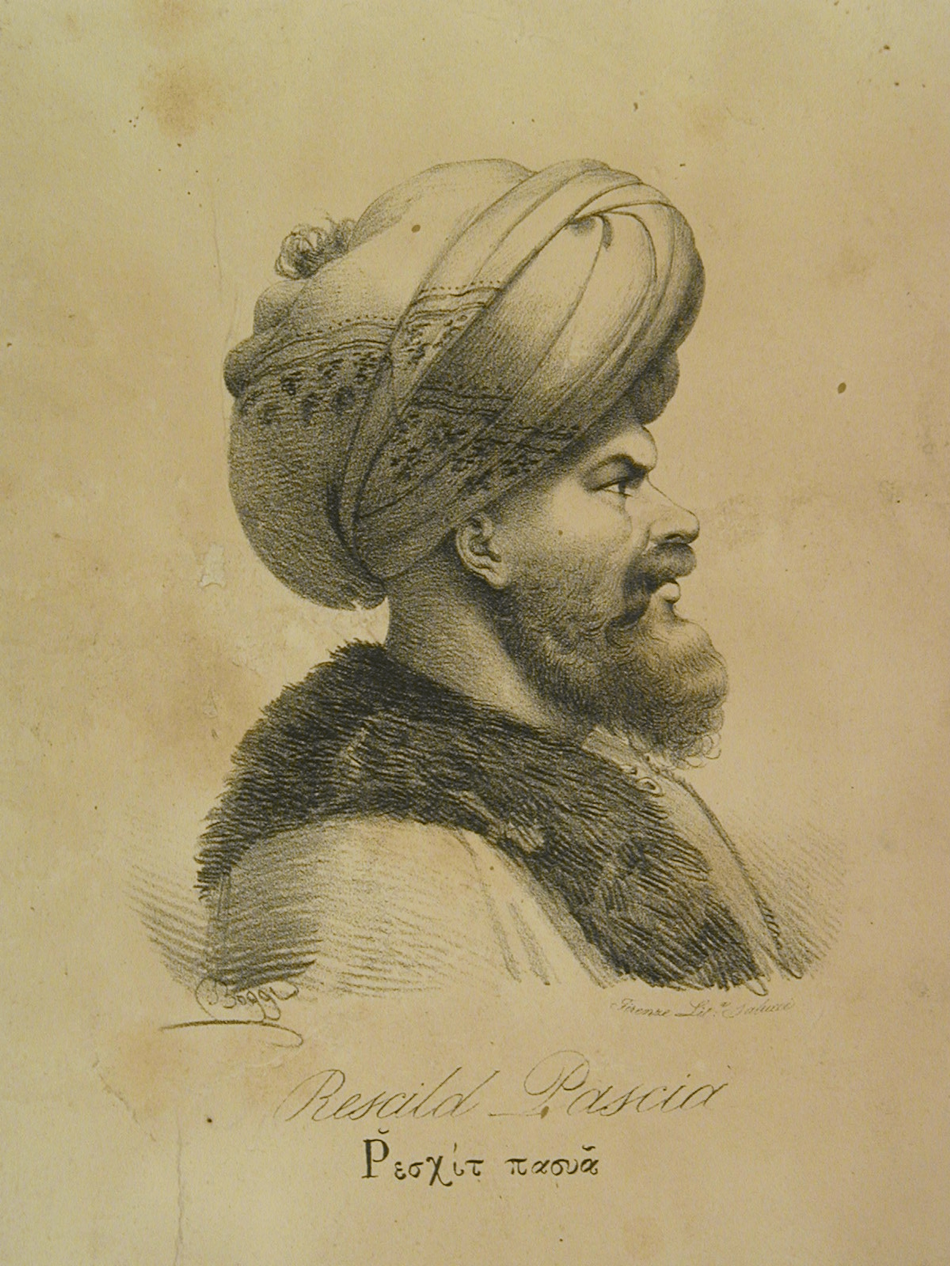
Potrait of Reşid Mehmed Pasha Kütahı by Giovanni Boggi Image Source: https://www.europeana.eu; This is an open-source image reproduced under the Creative Commons license.

**Figure 4:**
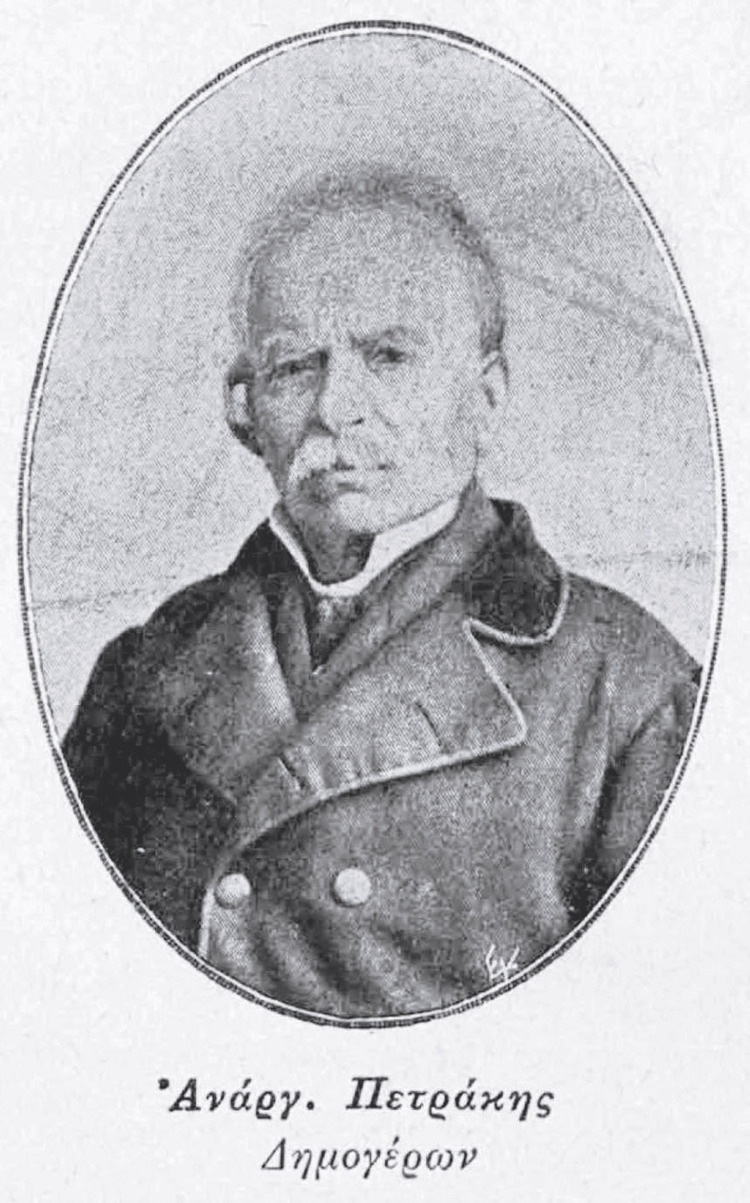
Anargyros Petrakis Image Source: https://www.elpis.gr; This is an open-source image reproduced under the Creative Commons license.

On December 1, 1834, the capital of Greece was transferred from Nafplio (Peloponnese) to Athens. However, the state of the city, especially after its destruction by Reşid Mehmed Pasha, also known as Kütahi (1780-1836) (Figure 3), was tragic [1,3]. In February 1834, Κing Otto (1815-1867) unofficially visited Athens, receiving an enthusiastic welcome and he was addressed by Anargyros Petrakis (Figure 4). Then, the foundation stone of the palaces was laid. The problem of cleanliness, directly accompanied by the health of the inhabitants, was severe. The city was hopelessly unclean and dirty. The streets, or the alleys, were full of dirt and stagnant water. There were several sewers and among them a central one, but this had suffered much destruction and had so many chasms that at every step there was a source of poisonous fumes [1].

In the elections of 1835, King Otto appointed Anargyros Petrakis, Μayor of Athens. Then, the Mayor and the Municipal Council began to work feverishly to deal with the acute problems of the Municipality, among which the health of the citizens had immediate priority since the swamp fever and cholera epidemics had reached uncontrollable proportions [1,2,4]. Among the general causes of the outbreak of the epidemics were the great humidity of the past spring, the great drought of the summer, as well as the frequent temperature changes. On the other hand, among the local factors for the increase in cases of swamp fever and cholera, the stagnant waters, the impurity of the ruins, the sewers, and the poor construction of the aqueducts should be considered [5,6].

It was one of the first major Asian cholera epidemics, which first hit Europe in the 19th century. The municipal authorities proceeded to pave the streets so that the impure waters would not pool and the swarms of mosquitoes that transmitted malaria would be removed. Nevertheless, the interest of the municipal authorities waned when the epidemic stopped. At that time, hospital treatment, in the sense of a responsible organization, was non-existent. The outbreak of epidemics led to a revolt of public opinion as the fear of the emergence of new diseases prevailed everywhere, with the increasing concentration of population in the urban centers. This created a social context within which the decision to establish a hospital was made [1].

In 1835, Ioannis Kolettis (1773-1847), physician and Secretary of the Interior, proposed to King Otto to establish a temporary hospital in Athens, the new capital. Immediately, the Prefect of Athens received a royal order to remove the soldiers stationed in the mosque, which was located near the wheat market, and to convert it into a temporary hospital [1,3]. In fact, it was an initiative of the Mayor, Doctor Petrakis, just like the other hygiene measures in the city. Mayor Anargyros Petrakis, after receiving the approval of Parish Councilors and Municipal Councilors, proceeded to implement the idea of ​​establishing a hospital. He addressed an appeal to all Greeks on January 7, 1836, asking for their assistance in establishing a municipal hospital in Athens [1,3]. The foundation stone of the Municipal Hospital of Athens was laid in June 1836 at 50 Akadimias Street, where today the Cultural Center of the Municipality of Athens is housed.

Among the first donors was the king of Bavaria Louis I (1825-1848), King Otto’s father, who offered 65,000 phoenixes, equivalent to 60,450 drachmas. Another great donor was Baron Konstantinos Vellios or Bellios (1772-1938), a prominent Greek merchant who lived in Vienna. He donated all his property in Attica, which amounted to 50,000 drachmas. Subsequently, other prominent personalities contributed to the construction of the Hospital, such as the diplomat and politician Alexandros Mavrokordatos (1791-1865), Sophie de Marbois-Lebrun, known as the Duchess of Placentia (1785-1854), I. Kontogiannakis (1817-1888), honorary Consul General of Greece in Russia, and Rallou Mourouzi (1782-1860), wife of the Phanariot prince, Konstantinos Mourouzis (1786-1821) [1,3].

For the construction of the Hospital, it was necessary to find and purchase the appropriate plot of land, which had to be located on the city’s outskirts. In a letter to King Otto, Drosos Mansolas (1789-1860), Minister of the Interior of Greece, presented five suggested locations: (i) The plot of Agia Sion, which was located within a built-up part of the city. It was not exposed to the north wind and its soil was stony. Due to the absence in the subsoil of residues from older constructions, there was no fear of unsanitary conditions; (ii) The plot of the Church of St. Philippou. However, it was located in the least hygienic part of Athens; (iii) The plot of the Agora seemed suitable due to its elevated location. Because it was very close to the center of the city and since the civil hospital must have a mobile unit, if it were located near the city, it would have offered great advantages to the patients; (iv) The plot behind the Acropolis, which was very close to the military hospital. However, this plot had some drawbacks. First of all, the hospital would have received the morning heat reflection from the rocks of the Acropolis and the midday winds. In addition, the military hospital was already located in that area and there were many remains of buildings in this place, requiring very deep foundations. Also, the civil hospital, which would have a medical clinic, must be adjacent to the University (the establishment of the latter had already been determined at the other edge of the city); (v) The plot where the first stone of the royal palace was laid. However, it was located far from the city and was exposed to the north wind. In addition, it was located on Areios Pagos Street, an area with unsanitary conditions [1].

After the pre-approval by the Royal Medical Conference (corresponding to today’s Ministry of Health), the Municipality of Athens decided to buy the plot of land in Agia Sion, the name of which came from the eponymous, older Christian church [7]. The plot was located on Akadimias Street, opposite Marseilles Street, and had an area of ​​10 acres. The first civilian hospital in Greece was to be built there. The Ephorate of the Municipal Hospital of Athens, whose members were the doctors Petros Ipitis (1795-1861), Nikolaos Kostis (1805-1861), Spyridon Patousas, alderman of the city of Athens, Andreas Kobatis, advisor to the Municipality of Athens and Dimitrios Misaraliotis, municipal councilor, signed the contracts for the purchase of the plot [2].

On May 18, 1836, the foundation stone for the construction of the Hospital was laid. The Municipality of Athens temporarily rented some buildings for the hospital treatment of patients until the building was completed in 1841, in an architectural design by Friedrich Stauffert, with some modifications by Edward Schaubert (1804-1860). Importance was given to the health issue when it was discovered that in 1841, out of 530 deceased individuals, only 130 had received medical treatment. Consequently, it was decided to appoint two municipal doctors to visit patients in need. Simultaneously, all necessary annexes were constructed. However, the financial situation of the Municipality had seriously deteriorated. Significant sums were spent in 1843 to address the emergency caused by a locust infestation, which severely damaged the crops. It was deemed necessary to appoint three municipal doctors to care for the large number of needy individuals afflicted by serious and now endemic diseases, such as typhus [1,3,8].

Despite all the difficulties, the hospital started operations a year later, in 1842. It was the first public civil hospital in Athens. On April 11, 1842, the Municipal Council appointed the first Board of Management consisting of: Bernard Reser (King’s Otto chief physician), Dimitrios Kallifronas (politician, 1800-1897), I. Klados (medical doctor), and Spyridon Paleologos. The respective Mayor, in this case, Anargyros Petrakis, would be appointed as President [1,9].

The Hospital had two clinics, a surgical one headed by Professor Ioannis Vouros (1808-1885) and an internal medicine one headed by Ioannis Olympios (1802-1869) [3]. It was initially constructed as a mezzanine building, with only the central section completed. In 1856, two additional wings were built on either side during a new construction phase. From the outset, it was designated as “Political” to differentiate it from the military structure, which had been erected under the Acropolis, at the foot of the southern side of the Holy Rock, opposite the ancient Asklepieion [1].

The Municipal Authority promptly arranged for the transfer of patients from the rented, unsuitable buildings to the rooms of the new facility. However, the operation of the new building placed certain administrative and financial obligations on the Municipality. Adhering to the legislation and addressing the urgent needs of the hospital, the Municipal Authority swiftly adapted to these requirements. It first focused on the administration and supervision of the new hospital following the law [1].

A clear example of the importance the Municipality placed on the smooth operation of its hospital was seen in the year 1840. Despite adverse conditions and a budget of approximately 153,000 drachmas, the Municipality did not hesitate to allocate nearly 10,000 drachmas for the institution’s needs [1]. In 1847, the Hospital’s budget was 10,000 drachmas out of the total Municipality of Athens budget of 144,000 drachmas. In October, a smallpox epidemic broke out in Athens, highlighting not only the crucial role of the Hospital but also its significant shortcomings [1,3].

Between 1848 and 1849, an internal medicine and a surgical clinic were established to meet the educational needs of the newly founded School of Medicine at the University of Athens (1837). In March 1849, a municipal tax of 1% was imposed “on goods and cereals imported into the district of the Municipality for internal consumption.” The proceeds were designated to meet the hospital’s various needs. In the same year, a new smallpox epidemic broke out. Later, in 1852, this tax would be extended to other products, such as tobacco and soap products, bringing in 66,750 drachmas for the Hospital [1,3].

During the Crimean War (1853-1856), a cholera epidemic broke out, starting from the island of Syros, a major center of maritime trade at that time [1,3,10]. The Greek Government and the Municipality of Athens quickly implemented all necessary measures to isolate the port of Piraeus. However, when these measures were eased, a cholera epidemic swept through Athens, reaching a critical level in October 1854, with thousands of people falling victim to the disease. The Municipal Hospital was soon overwhelmed with patients, but the staff and resources were insufficient to handle the crisis [3]. In 1857, the Mayor of Athens, Konstantinos Galatis (unknown-1857), donated his two buildings on Ermou Street to the Municipal Hospital, the Municipal Nursery, and the University of Athens [1,3].

Three years later, in 1860, a decision was made to construct a new wing with a total budget of 30,000 drachmas. Of this amount, 15,000 drachmas were allocated from the municipal budget, while the remaining 15,000 drachmas came from a donation by Queen Amalia (1818-1875). As a result, the first and second floors of the Hospital's east wing were completed [3].

In 1870, a special Royal Decree mandated that one-fourth of the 2% duty imposed on grain goods for internal consumption in the Municipality of Athens would be exclusively allocated to maintain the Elpis Municipal Hospital. The revenue from this tax was utilized to renovate the Hospital’s facilities between 1871 and 1875. Among other improvements, the old wooden beds were replaced with iron ones, and new bedding and nurses’ uniforms were purchased. Furthermore, two new toilets and a kitchen were constructed. The following year, in 1876, the construction of the western wing of the Hospital was completed [3].

A significant concern for the Municipal Authority was the city’s water supply. Since the severe shortage, the most pressing health issue for citizens was the quality of the water. The Hospital was overwhelmed with cases of enteric and typhoid fever, and the typhoid epidemic of 1881 claimed over a thousand lives in a short period. Dysentery and typhoid fever afflicted Athens throughout the 19th century and into the early decades of the 20th century, largely due to inadequate water supply and faulty drainage systems [1]. The stone water pipes not only caused substantial water loss but also frequently led to contamination from impurities. During Panagis Kyriakos’s (1829-1901) tenure as Mayor, from 1870 to 1879, substantial efforts were made to address significant challenges. Notable projects from this period include the construction of a permanent metal tank (the Lycabettus aqueduct), the doubling of street lamps, and the development of an extensive network of metal water distribution pipes [1].

In 1887, Theodoros Papapetrou was declared a benefactor of the nursing institution for his donation of 10,000 drachmas. In the same year, the Hospital acquired 100 garments for its patients for 8.10 drachmas each. Additionally, following the order of Professor Theodoros Aretaios (1829-1893), surgical tools worth 600 gold pounds and medicines worth 3,431 gold pounds were purchased. Despite these acquisitions, the Hospital faced a shortage of space due to the presence of patients with incurable diseases and abandoned individuals. To address this, the construction of a facility for incurable diseases was proposed, as well as transferring elderly and needy citizens to the poorhouse of the Municipality of Athens. Moreover, Professor Konstantinos Diligiannis (1833-1898), from the internal medicine clinic, suggested building three or four rooms on the second floor of the Hospital exclusively for tuberculosis patients. However, this proposal was not accepted by the Board of Management [1,3].

Nevertheless, in 1888, Spyridon Mercouris (1856-1939), the new director of the Hospital and future mayor of Athens, ordered the establishment of a special annex within the Hospital for patients with tuberculosis. This project, which cost 20,000 drachmas, sparked significant opposition due to residents’ fears about the spread of infectious diseases. At that time, the Hospital’s nursing staff was divided into two categories: Category A, which had four positions with a monthly salary of 60 drachmas, and Category B, with a monthly salary of 30 drachmas. Most of the nursing staff were men who lacked specialized nursing knowledge. Those in Category B could join without qualifications if they had served in the army, while Category A was reserved for individuals with additional relevant certifications or who had distinguished themselves in Category B [3].

Between 1887 and 1892, the Hospital was bolstered with additional resources, including bed linens donated by the Red Cross. The electrification process began as well as the facility’s capacity reached 140 beds, and 17 nurses across three categories were employed. It was also decided to install electric bells. Another significant project was the watercolor and oil painting of the Hospital. The demands of the Greek-Turkish war in 1897, though, transformed the nursing institution into a temporary infirmary for the wounded, who were cared for by volunteers and donors. This situation prompted the decision to construct a new, modern municipal hospital, while also leading to the approval of a proposal to provide care for private patients for a fee [1,3].

Elpis Hospital in the first half of the 20th century

In 1904, under the initiative of the Mayor of Athens, Spyridon Merkouris, a committee was established to explore the possibility of constructing a new building complex for the Municipal Hospital. The plan proposed building the new facility on a municipal plot in Ampelokipi, which was acquired in 1878. Initially, 55 acres of the 300-acre plot were allocated for this purpose, but this was later reduced to 18 acres [1,9].

The foundation stone for the new Municipal Hospital, Elpis, was laid on June 28, 1904, in a grand ceremony attended by the royal family, ministers, senior clergy, university professors, and a large crowd. The plan was designed by the architect and professor of engineering, I. Kolliniatis (1857-1921). It provided for the creation of 11 two-story kiosks, with a capacity of 50 beds each. In addition, a provision had been made for a residential building for the hospital staff, for two buildings with a pathological and surgical department and four auditoriums, an isolation kiosk, laundries and disinfection areas, baths, a kitchen, an engine room, a mortuary as well as for storage areas for horses and carriages. Among other things, it was designed to have a system of electric lighting since the continuous increase in the cost of lighting gas was one of the serious issues that concerned the administration of the Hospital, water supply, drainage, and steam heating. At the same time, the construction of a Christian church was planned for the religious needs of the patients. The cost of the original plan reached 4,000,000 drachmas and aimed at the construction of a nursing institution according to French standards [1,3].

Between 1907 and 1908, the Municipality of Athens faced difficulties in conserving resources. As a result, they decided to sell the first hospital building on Academy Street for 1,300,000 drachmas. However, the sale was never completed. In contrast, K. Sevastopoulos, a Greek national benefactor, bequeathed 100,000 drachmas for the construction of a wing in the new hospital, with the stipulation that it be named after him [3]. In the years 1908-1909, approval was given for the installation of a mechanical kitchen, cooking utensils, washing machines, and the provision of 20 baths. Although the first four kiosks of the new institution were ready to accommodate patients, an additional 39,832 drachmas were still needed to complete projects such as electric lighting, telephone installation, and the finalization of the sewage network [2]. Between 1912 and 1913, the "New Municipal Hospital” in Ampelokipi was completed. However, due to the ongoing Balkan Wars, the Prime Minister of Greece, Eleftherios Venizelos (1864-1936), requested that the hospital be handed over to the Hellenic Red Cross for the treatment and care of war wounded. From that point until 1970, it served as a Military Hospital. Meanwhile, in 1913-1914, the old hospital building on Academy Street underwent a complete renovation [2,9,11].

The Asia Minor Catastrophe had a significant impact on the Hospital. In 1922, the facility was opened to war refugees, who were provided with free treatment despite the Hospital’s strained financial condition [3]. Regarding the works, only essential adjustments related to transportation and minor repairs of lesser significance were undertaken. The onset of the global economic crisis in 1929 further constrained the Hospital’s resources [1,11].

**Figure 5:**
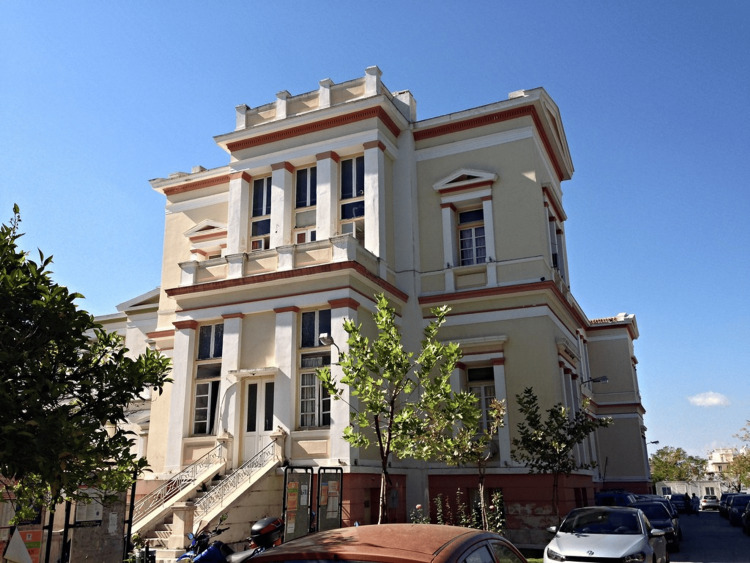
The Elpis General Hospital of Athens (2020) Source: https://www.elpis.gr; This is an open-source image reproduced under the Creative Commons license.

Almost 10 years later, in 1940, the new Mayor of Athens, K. Kotzias (1892-1951), scaled back the municipal authority’s plans to build a new hospital. Instead, a proposal was made to relocate the Municipal Hospital to the Semiramis hotel in Kifissia, but this decision was eventually postponed. Ultimately, the Pendelikon hotel was chosen for the relocation. The surgical, microbiological, and radiological departments, pharmacy, and financial services were moved to this new location. In contrast, the ophthalmology, otolaryngology, and dental departments remained at the Academy Street building [3]. During the German Occupation (1941-1944), the institution’s problems with food supply worsened after the Hellenic Red Cross decided to halt the distribution of medicines and food in 1942, due to a lack of supplies [12]. Following the withdrawal of the German and Italian occupying forces, an extensive renovation of the building on Akadimias Street was initiated. However, the Hospital struggled with financial and administrative challenges due to the unstable political climate and ongoing civil strife (1946-1948). In 1953, the Board of Management declined the proposal to integrate the Hospital into the Ministry of Welfare. For two decades, efforts were consistently made to relocate the Hospital from Akadimias Street. In 1971, the building on Alexandra Street was no longer used as a Military Hospital and began serving its originally intended purpose as the Municipal Hospital of Athens. Since 1983, it has been part of Greece’s National Health System, providing comprehensive care (Figure 5) [3]. As of 2024, it offers a range of services, including emergency care, general surgery, internal medicine, and specialized care in various medical fields. Like other public hospitals in Greece, it faces financial challenges, particularly due to budget constraints in the healthcare sector.

## Conclusions

The Municipal Hospital of Athens was the first civilian and university hospital in the Greek capital. From its inception, it operated on a charitable rather than profit-making basis. The administration and financial responsibility of the hospital were entrusted solely to the Municipality of Athens, with the state supporting it only through legislative measures and resource taxation in its favor. The Hospital’s history is deeply intertwined with the development of Athens as the capital of Greece, and it is closely linked to the National and Kapodistrian University of Athens. Renowned doctors practiced there, and the hospital played a significant role in advancing scientific medicine. Between 2002 and 2010, the hospital underwent extensive repairs, maintenance, and expansion. The renovations focused primarily on the interiors, including the construction of intermediate levels to subdivide the particularly high ceilings of the interior spaces.
